# Environmental and socio-economic impacts of new plant breeding technologies: A case study of root chicory for inulin production

**DOI:** 10.3389/fgeed.2022.919392

**Published:** 2022-10-06

**Authors:** Maria Hingsamer, Veronika Kulmer, Matthew de Roode, Michael Kernitzkyi

**Affiliations:** ^1^ Joanneum Research Forschungsgesellschaft mbH, LIFE—Institute for Climate, Energy and Society, Graz, Austria; ^2^ Sensus BV, Roosendaal, Netherlands

**Keywords:** LCA, life cycle assessment, socio-economic assessment, inulin, chicory, new plant breeding, GHG emissions, value added

## Abstract

In Europe, root chicory and other plants are cultivated for their prebiotic food fiber, inulin, which boosts the growth of beneficial gut bacteria and stimulates the human immune system. CHIC, a H2020 project, develops new chicory variants which produce more and reported to be healthier inulin as well as medicinal terpenes. This paper presents an environmental and socio-economic assessment of the whole value chain of the new chicory variants and their derived products using a case study based in the Netherlands. Two scenarios based on new chicory variants using new plant breeding technologies (NPBT) are analyzed and impacts thereof are compared to the reference scenario; the current commercial inulin process from conventional chicory. Both scenarios show higher inulin content, but the inulin adsorption process differs. While one aims to optimize inulin yield, the other one explores the potential of a multipurpose use, yielding inulin and health beneficial terpenes. Methodologically, we employ multi-regional input-output (MRIO) analysis to estimate additional economic benefits, added value and job creation, while by means of life cycle assessment (LCA) effects on greenhouse gas (GHG) emissions and primary energy demand are derived. Both methods, MRIO and LCA, are well suited to analyze the raised issues and draw on the same data. Generally, the results highlight the importance of inulin production at a national and EU-level in the reference scenario. In case of the two scenarios, we find that the related socio-economic impacts are much higher than in the reference scenario and thus highlight their ability to boost economic activity and increase competiveness of the EU, i.e. over 80% of the generated value added stays in the EU. In terms of environmental impacts, the two scenarios show lower GHG emissions and primary energy demand due to the higher efficiencies of the process in the scenarios compared to the reference inulin process. Additionally, regarding the goal of climate neutral production, we find that the majority of GHG emissions stem from the electricity mix and natural gas demand. Replacing these sources of energy with more renewable ones will contribute to this goal.

## Introduction

The two cornerstones of, the European Green Deal, Farm2Fork (COM 2020a) and the Biodiversity strategy (COM 2020b), provide the goal of a fair, sustainable, healthy and environmentally friendly food system in Europe. In the future, the food system and food supply is going to face a variety of challenges such as climate change induced increase of adverse weather conditions as well as threats to plant health, environmental pollution, rising land consumption as well as deforestation and resource dependence abroad (IPCC, 2022). Plant breeding is one strategy to achieve the long-run goal of a sustainable, robust and resilient European food system. In the past, innovations in plant breeding led to an increase in the average yield per hectare and hence brought positive economic, social and environmental effects (Noleppa and Cartsburg 2021; Smith et al., 2021, Jones et al., 2017). However, it is evident that conventional breeding approaches restrict the number of crop improvements due to their limited genetic diversity. New plant breeding technologies (NPBTs), have rapidly emerged in recent years, due to their ability to accelerate crop improvement through the introduction of genetic variation in a targeted manner (Ricroch 2019; Ricroch et al., 2022). These NPBTs drive the development of traits in new crops and allow for biofortification, yield improvements, and enhanced pest and disease resistance, adaptation to climate change, and result in new industrial and pharmaceutical applications (Ricroch 2019).

In the H2020 CHIC project (H2020-NMBP-BIOTEC-07-2017, GA No. 760891), NPBTs were implemented in root chicory, in order to establish it as a multipurpose crop and as a sustainable approach to produce dietary fiber, inulin, with enhanced prebiotic effects that promote gut health. Drawing on the new variants generated in the CHIC project (Matos et al., 2020; Baixinho et al., 2021; Cankar et al., 2021; Häkkinnen et al., 2021; Matos et al., 2021), and their bioactive characteristics, this paper assesses the socio-economic and environmental impacts of the new chicory variants using the case study site of Sensus BV production plant, located in the Netherlands. More precisely, the socio-economic assessment quantifies production, output of goods and services, value added and number of jobs, while the environmental assessment calculates the resulting greenhouse gas (GHG) emissions and cumulated primary energy demand. In doing so, this study extends the literature twofold: First, we enrich the debate on effects of dietary fiber by assessing socio-economic and environmental impacts. Second, we show how the application of new plant breeding technologies influences these impacts.

Root chicory (*Cichorium intybus* var. *sativum*) is an herbaceous plant with a fleshy taproot that can grow up to 75 cm in length (Baert and Van Bocksdaele 1992; Street et al., 2013; Häkkinen et al., 2021). It is predominantly grown in the North-West of Europe and is the main source of the dietary fiber inulin (Van Arkel et al., 2013). The taproot contains an average inulin content of 17% by fresh weight and a typical root yield is of 45 tons per hectare (Wilson et al., 2004). Currently the global acreage of root chicory amounts to around 14,500 ha (FAOStat 2017). Root chicory also contains normally a large amount of sesquiterpene lactones (STLs) (Cankar et al., 2021). Some of them show medicinal properties such as anti-inflammatory, anti-cancer and analgesic activity and therefore these ingredients are studied in medical applications (Chadwick et al., 2013). Consequently, due to its biosynthetic capacity, high yields and low agronomic requirements, chicory has high potential to become a versatile production host for molecular farming by providing many additional health-related products (Meijer and Mathijssen 1992). These include immunomodulatory prebiotics (Roberfroid 2008) and pharmaceuticals to prevent lifestyle diseases [e.g., obesity (Antal et al., 2008)], to promote gut health, to stimulate the immune system (Bodera 2008) or as alternative antibiotics (Sinkovic et al., 2017; Peña-Espinoza et al., 2018). To achieve this, new chicory variants need to be developed. However, chicory breeding is currently exceptionally time-consuming (Baert and Van Bocksdaele 1992; Red'ko et al., 2008; Shoorideh et al., 2014). Since it is an obligatory outcrossing species, no true varieties can be obtained and germplasm is maintained by *in vitro* propagation. Therefore, chicory is a highly relevant case where new plant breeding technologies (NPBTs) can make a key difference.

Inulin is a natural, low-caloric value, water-soluble dietary fiber (see EC 1169/2011, Alinorm 09/3/12, March 2009) found in a variety of fruits, vegetables and herbs. The gut bacteria transforms inulin to short chain fatty acids contributing to local and whole body health (Ahmed and Rashid, 2019; Roberfroid 2008; Liu et al., 2022). Currently it is mainly applied as a food fiber supplement and low-calorie sweetener in various food products (e.g., dairy products, confectionary, infant food), but has gained increasingly in importance in cosmetic as well as pharmaceutical applications (for an overview see Cankar et al., 2021). Inulin affects physiological and biochemical processes, which leads to positive health impacts and reduces the risk of many diseases (Street et al., 2013). The inulin market size is well over 200 kilo tons, with a global market volume of around 1 billion USD in 2021 (Market research international, 2022). The three biggest exporters of inulin are Belgium, Chile and the Netherlands with a total share of around 99% of the total exports (BACI database, 2020).

To the best of our knowledge, studies analyzing socio-economic and environmental impacts of nutrient fibers, even based on conventional breeding technologies, are scarce. Although the debate on human health effects of dietary fiber such as inulin has greatly increased in importance in the recent years (see Dahl and Stewart 2015; Zhao et al., 2018; He et al., 2022), the resulting socio-economic and environmental implications are still under-researched. The main reason is the lack of data availability and insufficient data quality (Gomesz-Barbero et al., 2008; Smale et al., 2009). In order to assess the socio-economic and environmental implications of dietary fiber, the whole value chain, including every production stage, from cradle, i.e. cultivation to gate, i.e., industrial processing, has to be analyzed.

Still, the evaluation of socio-economic and environmental impacts of bio-based innovations are frequently applied in the fields of bioenergy, biomaterials, biochemicals and the food sector. Applications of bio-based innovations are various and comprise biomass-based energy (Van Dam et al., 2010; de la Rúa and Lechón 2016; He et al., 2016; Perrin et al., 2017; Weik et al., 2019; Lozano Miralles et al., 2020; Auer and Rauch 2021.), biofuels (De Carvalho et al., 2016; Ji and Long 2016; Lechón et al., 2019; Jeswani et al., 2020; Wang et al., 2020) and biomass-based materials and chemicals (Valente et al., 2018; Ruiz Pachón et al., 2020). Assessments in the food sector focus on crops as well as crop-based products (Notarnicola et al., 2017; Cederberg et al., 2019; Kulmer et al., 2020; Garcia Gonzales and Bjornsson 2022), milk and milk based products (Revéret et al., 2015; Romano et al., 2021), vegetables as well as meat (Torrellas et al., 2012; Mugumaarhahama et al., 2021; Wilfart et al., 2021; López-Andrés et al., 2018) and food production in general (Gunasekera and Finnigan 2010).

The paper is organized as follows: we describe materials and method in Section Materials and methods. Section Results reports the results for the socio-economic and environmental impact assessment, while we discuss our main findings and draw conclusions in Section Discussion and conclusion.

## Materials and methods

In this section, we provide details on the methodologies used for the socio-economic and environmental impact assessment; the multi-regional input-output model is used for the socio-economic impact assessment and the life cycle assessment is used to assess environmental impacts. This is followed by a detailed description of the different scenarios and the case-study site.

### Multi-regional input-output model

Methodologically socio-impact assessments mainly use multi-regional input-output analysis (for an overview see Brinkman et al., 2019), because they can differentiate between direct, indirect, and induced effects and can include multiple impacts in contrast to computable general equilibrium (CGE) and partial equilibrium (PE) models. MRIO models have the ability to trace the whole production process from its origin *via* intermediate production stages to its final destination. The tracing starts with primary inputs like labor, capital, land or other environmental goods and ends at the final consumption of produced goods and services (Steen-Olsen et al., 2012; Weinzettel and Wood, 2018; Kulmer et al., 2020). Due to the interregional interlinkages, regions where the effects materialize (e.g., where value added is created) and activities that generate economic activity elsewhere (i.e. cause the largest spill-over effect) are easily identified (Lenzen et al., 2012; Giljum et al., 2015).

MRIO models are interconnected linear equations that represent, commonly in monetary values, the flows within the global economy. We can use the matrix notation to describe the different elements of the equations system and how the total output of the global economy is either used by industries as intermediate inputs, or to satisfy final demand. In order to enhance readability and clarity for the reader we abstract from a sector disaggregation:

$$\left[\begin{array}{c} X_{1}\\ X_{2}\\ \vdots \\ X_{R} \end{array}\right]=\left[\begin{array}{cccc} A_{11} & A_{12} & \cdots & A_{1R}\\ A_{21} & A_{21} & \cdots & A_{2R}\\ \vdots & \vdots & \ddots & \vdots \\ A_{R1} & A_{R2} & \cdots & A_{RR} \end{array}\right]*\left[\begin{array}{c} X_{1}\\ X_{2}\\ \vdots \\ X_{R} \end{array}\right]+\left[\begin{array}{c} Y_{1}\\ Y_{2}\\ \vdots \\ Y_{R} \end{array}\right]$$ (1)

$X_{i}$ denotes the column vector of total output by economic sector in country *i* (i,j∈R) . *A* is a coefficient matrix describing input per output ratios in the production of these sectors with $A_{ij}$ denoting inputs from sectors in country *i* required to produce one unit of output from each sector in country *j*. $Y_{i}$ is a column vector of total final demand for the output of country *i*.

This equation can be transformed into $X={\left(I-A\right)}^{-1}Y$ , where ${\left(I-A\right)}^{-1}$ describes the Leontief inverse matrix L , which represent the output multipliers in relation to final demand. Given any final demand, the total output needed to meet that demand is simply the product of the Leontief inverse matrix, and the final demand. That is the direct and indirect requirements per unit of final demand.

Through the MRIO analysis, we can estimate the total output in monetary terms that will be produced by the different sectors in the economy in order to satisfy the intermediate and final demand of goods and services. Eq. 1 can be also decomposed as follows:

$$X=\left(I+A+AA+AAA+\ldots +A^{n}\right)y$$ (2)

where Iy are the direct impacts and $\left(Ay+AAy+\ldots +A^{n}y\right)$ are the indirect as well as induced impacts. The value added multiplier is given by vL , where the vector v=V/X and where the vector V represents total value added from countries and industries. Analogously, the employment multiplier is given by eL , where the vector e=E/X and where the vector *E* represents employment by sector and country.

The MRIO model is calibrated to the EXIOBASE version 3 data (Wood et al., 2015; Stadler et al., 2018) of 2019. The model comprises 163 industries and covers 44 countries and five rest of the world regions by continents. From the several MRIO databases such as WIOD, EXIOBASE, EORA, OECD Inter-Country Input-Output and GTAP available to conduct global analysis we choose EXIOBASE based on the following criteria: 1) high resolution of CHIC relevant sectors such as numerous agricultural activities, food sectors, food processing industries and pharmaceutical, 2) number of regions and countries depicted, 3) extensive satellite account of environmental, economic and agricultural aspects as well as 4) most recent dataset with a consistent annual global MRIO tables for the time span 1995–2019 (note that from 2011 on the MRIO tables are projections).

### Life cycle assessment

We apply the method of an attributional “Life Cycle Assessment (LCA)” to assess environmental impacts of a product. LCA has become a standard methodology to assess environmental impacts, defined in the International Standards ISO 14040 (International Organization for Standardization, 2006): LCA is a method to compile and assess the input and output flows as well as the potential environmental impacts of a product system during the various stages of its life cycle. Environmental impacts include the use of natural resources and the effects of emissions. The stages include extraction of raw materials, manufacturing, distribution, product use, recycling and final disposal (from cradle to grave/gate) (ISO 14040, International Organization for Standardization, 2006). In this study, we apply a “cradle to gate” assessment and the impacts focused on are greenhouse gas (GHG) emissions and primary energy demand.

Methodologically, an LCA is structured in four consecutive stages: 1) goal and scope definition (including a clear definition of the functional unit, system boundaries—see Figure 2—and associated assumptions); 2) life cycle inventory (the compilation of all the inputs and outputs respectively from and to nature associated to all processes that form part of the system’s life cycle); 3) life cycle impact assessment (in which the full inventory of inputs and outputs is translated into a number of aggregated metrics of environmental impacts); and 4) interpretation (in which results are discussed and compared to suitable benchmarks) (for details see ISO 14040, International Organization for Standardization, 2006).

For the assessment of the contribution of the GHG emissions, the global warming potential on 100-year time horizon (GWP 100) is used. The GHG—CO_2_, CH_4_, N_2_O—are expressed in terms of equivalent amount of CO_2_ (CO_2_-eq). Therefore, the CO_2_-eq factors are taken from (Myhre et al., 2013) using the factors including climate carbon feedback. Direct and indirect emissions are included in the assessment. CO_2_ emissions from burning biomass are balanced zero according to IPCC guidelines (IPCC, 2019). The cumulated primary energy demand (in MWh) includes the total energy demand (fossil, renewable, other) of all process steps of the inulin production.

Two types of data are used in the LCA calculation—namely foreground and background data. Foreground data is mainly based on the information collected from the case study site by Sensus BV; details are provided in Section Scenarios. Background data for materials, fuels and transport is mainly gathered from the database Ecoinvent 3.7.1 (Wernet et al., 2016), for some information, GEMIS 5.0 (IINAS 2021) is used. Information on the electricity mix for the Netherlands is drawn from European Commission (2020).

Both assessments—the MRIO model and LCA—use a production-based approach and build on the same assumptions, allowing a consistent combined analysis of economic and environmental impacts. Both approaches evaluate the whole upstream supply chain.

### Scenarios

**FIGURE 1:**
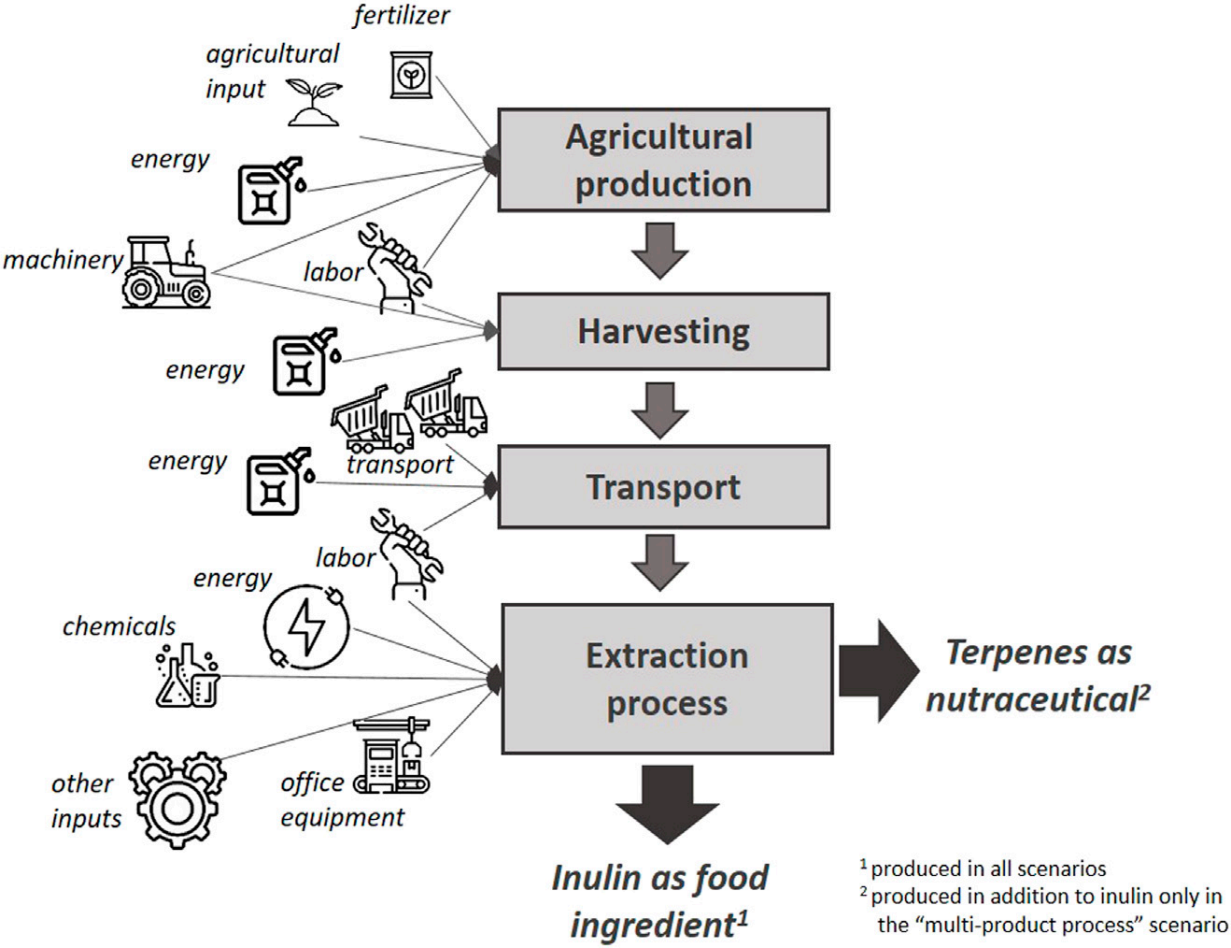
Activities included in the analysis–simplified scheme.

In this paper, we study the whole value chain of chicory based inulin with all production stages (Figure 1) based on the case study site; the production plant of Sensus BV., located in the Netherlands. In general, chicory roots are harvested from September to December (in the northern hemisphere and depending on the local weather conditions) (Gordon et al., 2018). The harvested roots are then transported to the factory, where they are washed, chipped and cleaned. Next, the roots are extracted with hot water, after which subsequent purification steps are applied to remove proteins, minerals, polyphenols, organic acids and sesquiterpene lactones (herein after “terpenes”). The type of purification and the order of process steps may differ but ultimately, the liquids in the purified process stream are evaporated and spray dried to yield pure inulin (Paine et al., 1932; Leclercq and Hageman 1993; De Leenheer 1996; De Leenheer and Smits 1999; Oliveira and Park, 2008). The common denominator of all current commercial inulin processes is that the processes are devoted to the purification of inulin without a lot of focus on the purification of other potentially valuable components. Of course, side streams are processed in the most optimal and sustainable way.

The current status quo of inulin production from conventional chicory serves as reference scenario and describes the current commercialized process. Two NPBT scenarios are analyzed and impacts thereof are compared to the reference inulin process. The scenario “Improved inulin process” denotes NPBT chicory with a higher inulin content, hence enabling a higher yield of inulin compared to the reference scenario. Although the process choice is similar to the current inulin production process, the reduction of terpenes in the NPBT chicory variants results in a reduction of required purification steps with respect to the current process. It must be understood that the breeding results from the CHIC project did not result in a chicory variant with a higher inulin accumulation per plant. The resulting variant appeared to have a downregulated stress reaction to cold and folium reduction. In the reference case, chicory will breakdown inulin under typical seasonal stress conditions that occur during autumn (increasing cold periods and degradation of leaves). Logistically, this period is roughly in the middle of the harvesting period of the inulin campaign and, thus, has an impact on the yearly overall inulin content available for extraction. By reducing this stress reaction, the average reference inulin level will be retained longer during the harvesting period and thus result in an overall higher content and yield of inulin per year. For comparison reasons, this overall higher inulin content has been represented in a percentual increase per plant in the scenario calculations (19% instead of 17%). The scenario “Multi-product process” denotes a multi-ingredient process, where NPBT chicory has not only a higher inulin content but also includes specific health beneficial terpenes. Thus, in a different adsorption process, two products are yielded, inulin (same amount and quality as in the improved inulin process) and terpenes (applied as nutraceuticals). In general, optimizing a specific trait by breeding can lead to undesired tradeoffs on other aspects. In both scenarios that are presented in this paper, the results of the breeding experiments were grown in greenhouses to full plants. Although greenhouse conditions are different to open field conditions, the greenhouses were by no means sterile environments but required given the current regulatory status of the NPBT techniques that were used. The fact that fully grown plant could be harvested gave us confidence that these traits can result in a viable commercial crop.

Supplementary Appendix Table SA1 provides information on the characteristics of the assessed scenarios. It is assumed, that the chicory variants in the two NPBT scenarios are not considered genetically modified organisms (GMO), due to the European Court of Justice ruling of 2018 (European Court of Justice, 2018). When the new chicory variants are deemed to be GMO (which they currently are by ruling of the European Court of Justice), only a non-food value chain remains feasible since it is not foreseeable that food (supplement) applications on the European market are viable for ingredients from GMO crops.^1^

In order to compare the two NPBT scenarios to the reference scenario, the same set of assumptions regarding cultivation (open field cultivation on the case study area), annual average yield (46 t chicory per ha), market value of inulin (about 2 k € per t; product quality is equal to the current commercial product) and terpenes sold as a concentrated syrup (about 200 k € per t active ingredient) underlies the analysis. The use of fertilizer and pesticides per ha are assumed equal in both scenarios and the reference scenario. Due to the actual state of knowledge, it cannot be assumed that the new chicory variants need a higher or lower amount of nutrients or pesticides. The main difference between the NPBT scenarios and the reference inulin process is the inulin content of chicory roots. While in the reference scenario the average inulin content of 17% is assumed, the NPBT scenarios are characterized by a significantly higher inulin content of 19%. Consequently, on the one hand, production output in physical and monetary terms exceeds the one of the reference inulin process and on the other hand, we find higher efficiencies in terms of inulin yield per ha land. Costs and emissions of infrastructure construction such as buildings or large-scale machinery are irrelevant and thus not included in our assessment, as all scenarios show the same requirements. Additionally, the side stream chicory root pulp is not considered in our assessments due to the low economic value and hence negligible nature. Details on the economic and environmental performance of each scenario are illustrated in Section Economic and environmental performance of scenarios.

Data on farming of chicory and inulin as well as terpene production stems from Sensus BV the case study site of the CHIC project. Sensus BV is one of the largest manufacturers of chicory inulin and involved in the complete supply chain from farm to health promoting ingredient. Sensus BV provided data and assumptions on the current commercial process of inulin production as well as business cases based on modelled data on the inulin and terpene production of NPBT chicory variants. Furthermore, Sensus BV provided information on open field cultivation of chicory. Guidelines for farmers on cultivation of chicory in Netherlands and continental Europe (Wageningen University & Research (Ed.), 2018) complemented data of Sensus BV. Data on the bio-activity and hence on content of inulin and terpenes of chicory used to derive the business cases of the two NPBT scenarios stem from experiments in the CHIC project (Matos et al., 2020; Baixinho et al., 2021; Cankar et al., 2021; Häkkinen et al., 2021; Matos et al., 2021). Additionally we draw on pertinent studies to validate key assumptions on price of key parts, such as seeds, and productivity changes of NPBTs (Garcia-Yi et al., 2014; Noleppa 2016; Jones et al., 2017).

The MRIO analysis calculates the socio-economic impacts associated to a final demand of goods and services, denoted as *y* in the previous equations (Eq. 2). Thus, it is necessary to specify all goods required along the analyzed industrialized value chain and to identify the sectors that supply them in the economy to build the final demand vectors y (de la Rua and Lechón 2016; Wang et al., 2020). In this study, the final good is inulin and in one case inulin and terpenes, which we define by all goods and services necessary required to yield the respective final good (details are described in Section Economic and environmental performance of scenarios). This approach of tacking a step back and specifying the whole value chain is state-of-the art in MRIO analysis (Miller and Blair 2009).

**FIGURE 2:**
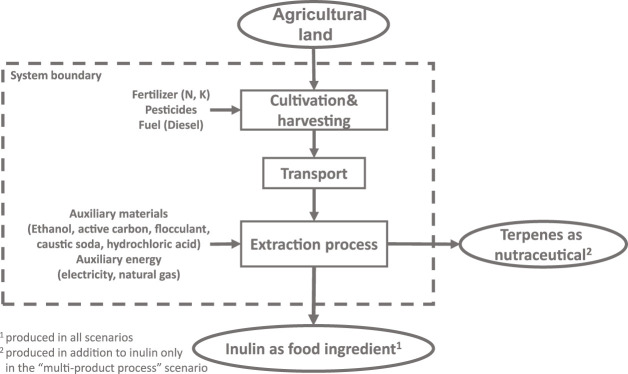
System boundary of the scenarios in the LCA.

Similarly, the goal of the LCA is to calculate the respective environmental impacts “from cradle to gate” (as mentioned also in Section Life cycle assessment) of the derived inulin product on a yearly basis and uses the functional unit of 1 t inulin leaving the production process. Therefore, the mass and energy balance of the whole value chain is needed to translate the yearly in- and outputs to environmental impacts. The system boundary (Figure 2) include the production of input substrates (e.g., fertilizers, pesticides) needed for cultivation and harvesting process, the transport to the production facility, the auxiliary materials and auxiliary energy needed in the production process. An average transport distance of approx. 100 km of the roots to the processing location is assumed. For comparison reason an allocation in the scenario “multi-product process” is needed. There are different allocation methods (see e.g., ISO 14040, International Organization for Standardization, 2006) that allocate GHG emissions and primary energy demand to the main and secondary products according to mass, energy content, exergy content and economic value added. Regarding the estimation of GHG emissions and primary energy demand, an allocation by mass in tons of the derived products inulin and terpenes is most suited, and the impacts are allocated to the amount of inulin produced.

## Results

The results section is structured as follows: First, we provide data and details on assumption underlying the socio-economic and environmental impact assessment. Second, the socio-economic impacts regarding production output of goods and services, value added and job creation for all scenarios with sectoral and regional details are illustrated. Third, in a similar vein, resulting environmental impacts regarding GHG emissions and the primary energy demand of the scenarios are discussed. Both assessments focus on the comparison of the effects of the two NPBT scenarios with the reference inulin process.

### Economic and environmental performance of scenarios

Based on the activities required along the whole process and the associated costs, we have calculated the economic performance of inulin and terpenes from chicory. Table 1 shows the aggregated costs of the whole value chain for each scenario (details are reported in Supplementary Appendix Table SA2). The final demand vector for each scenario describes the costs in monetary units required to cultivate, harvest, wash and chip chicory, its transport and storage as well as the final process in the factory to get inulin and terpenes. Table 1 reports the cost vectors, which are then used to calculate the socio-economic effects associated to the system. Note that, the socio-economic assessment assumes that in all three scenarios the same amount of chicory (i.e., 60,000 t) is harvested. Consequently, the higher inulin content of chicory in the two NPBT scenarios enables a higher inulin yield and hence higher production output compared to the reference inulin process.

**TABLE 1:** Cost vector of each scenario by sector aggregate.

	Reference inulin process		Improved inulin process		Multi-product process	
	M EUR	Cost share (%)	M EUR	Cost share (%)	M EUR	Cost share (%)
Agriculture	0.64	6	0.71	6	0.71	5
Manufacturing	1.08	10	1.15	10	1.57	11
Fossil fuels	0.25	2	0.26	2	0.53	4
Chemicals and plastics	1.45	13	1.49	13	1.90	13
Electricity	1.51	14	1.61	14	1.63	12
Trade and repair services	1.43	13	1.53	13	2.09	15
Transport	2.06	19	2.06	18	2.08	15
Other services	2.59	24	2.76	24	3.56	25
*Total final demand*	*11.01*		*11.56*		*14.07*	
*Direct value added*	*7.36*		*10.38*		*15.96*	
**Total market value**	**18.37**		**21.93**		**30.03**	

In total, more than twenty activity sectors provide the goods and services required directly by the final demand. Although the production value is significantly higher in the NPBT scenarios, the technology and hence the economic structure are similar in all scenarios. Manufacturing goods and chemicals account for almost a quarter of total costs, while electricity used in the process to generate inulin and terpenes accounts for about 12%–14%. Although the cost share of fossil fuels is quite small, the multi-product process requires twice the amount (4%). Transport from and to the factory and services such as administrative, business and renting services are substantial cost categories. Agriculture costs are a bit higher in the NPBT scenarios, since gene edited seeds are more expensive than its conventional equivalent. Furthermore, the NPBT scenarios generate a higher amount of direct value added (Table 1) compared to the reference inulin process, which is calculated by subtracting of intermediary inputs from total revenues. More precisely, in the NPBT scenarios direct value added makes up half of total production output (in the reference scenario it is only a third). One reason is that due to the higher inulin content of chicory roots, farmers receive a higher price compared to the reference inulin process. In the multi-product process, the higher direct value added compared to the improved and reference inulin process, traces back to 1) higher capital demand due to a different structure of the multi-ingredient process and 2) higher revenues for farmers and the factory, due to the additional market value of terpenes.

For the environmental assessment, the mass and energy balance of the different processes is most important. Table 2 reports the main specifics of each scenario. The NPBT chicory variants based scenarios yield higher outputs in terms of inulin produced per t chicory root, due to a higher content of inulin (19%). To compare thethree scenarios the same amount of inulin produced (10,619 t inulin) per year is applied in the LCA in line with the functional unit of 1 t inulin. In the socio-economic assessment the comparison of scenarios is based on 60,000 t of harvested chicory roots in each scenario.^2^ The heating value of inulin and the chicory roots is equal in all scenarios and hence excluded from the comparison. Furthermore, as before mentioned, we need an allocation to include the environmental impacts resulting from the amount of inulin produced in the multi-product process. For the reference and improved process there is only one product (inulin) and it is allocated 100% of the GHG emissions and primary energy demand for the process. For the multi-product process, since there are two products (inulin and terpenes) the emissions and primary energy demand must be allocated between to each product. By mass allocation (applied in this study, see also Section Scenarios) in terms of t inulin and terpenes, approx. 83% of the total GHG emissions and the total primary energy demand are allocated to the amount of inulin produced. If we would apply a mass allocation using t dry matter of products, approx. 91% of the total GHG emissions and the total primary energy demand would be allocated to the inulin produced. If we would use an economic allocation approx. 74% of the total GHG emissions and the total primary energy demand would be allocated to the produced inulin, assuming the same market prices as in the socio-economic assessment (for details see Section Scenarios). Part of the life cycle inventory is included in Supplementary Appendix Table SA3.

**TABLE 2:** Data on yield and energy demand of scenarios.

	Reference inulin process	Improved inulin process	Multi-product process
Inulin content [%]	17	19	19
Chicory roots [t/a]	67,288	60,000	60,000
Produced inulin [t/a]	10,619	10,619	10,619
Produced inulin [t_DM_/a]	10,300	10,300	10,300
Produced terpene mixture [t/a]	—	—	2,111
Produced terpene mixture [t_DM_/a]	—	—	1,056
Thereof active ingredients [t_DM_/a]	—	—	38
Natural gas demand [M NM^2^/a]	0.3	0.3	1.2
Electricity demand [GWh/a]	21	19	19

### Socio-economic impacts

Table 3 reports the production, value added and job generated of the global economy in order to satisfy the final demand of all goods and services required along all stages, from chicory sowing until the gate of Sensus BV to yield inulin and terpenes. As expected, due to the higher productivity of NPBTs, the socio-economic impacts are much higher than in the reference scenario; with the multi-product process showing the highest effects. In the latter the global economy produces about 74 M € of goods and services, from which almost 41 M € contribute to global GDP. Along with this increase in economic activity are positive employment effects with nearly 1,000 jobs. Socio-economic impacts of the reference inulin process and improved inulin process are similar regarding direction of effects, but on a smaller magnitude (two third of the multi-product process).

**TABLE 3:** Socio-economic effects of each scenario.

	Reference inulin process	Improved inulin process	Multi-product process
Total impact on			
Production of Good & Services (M EUR)	52	57	74
Value added (M EUR)	25	30	41
Jobs (number of persons)	652	746	991
Final demand multiplier			
Output multiplier	4.3	4.3	4.3
Value added multiplier	2.1	2.2	2.4
Employment multiplier	0.054	0.056	0.057

**FIGURE 3:**
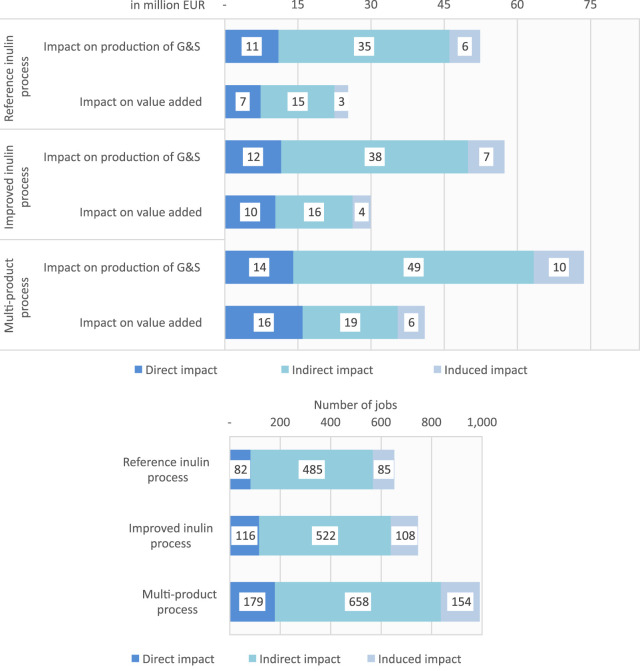
Direct, indirect and induced impacts per category (value added [M €], production of good and services (G&S) [M €], jobs [number]) and scenario.

Using MRIO analysis enables us to decompose the socio-economic impacts in three types of effects: 1) The direct effect measures economic activity directly associated with the production of goods and services related to our systems (inulin and terpenes from chicory). 2) The indirect component measures the production of goods and services of the backward-linked industries supplying the direct goods and services. 3) The induced component measures the effects of increased income of the direct employees (all employees associated directly along the value chain with the production of inulin and terpenes). Figure 3 shows importance of these three impacts in each socio-economic effect. As expected, the induced effects have the lowest contribution in all scenarios, while the indirect effects has the highest one. In the multi-product process, indirect impacts contribute over 49 M € to total effects, accounting to over 65%. In case of employment, the importance of the indirect effect is even more pronounced and it generates more than 70% of the impacts in all scenarios. Value added is the category, where direct impacts are the highest (with a contribution of 30%–35%, depending on the scenario).

**FIGURE 4:**
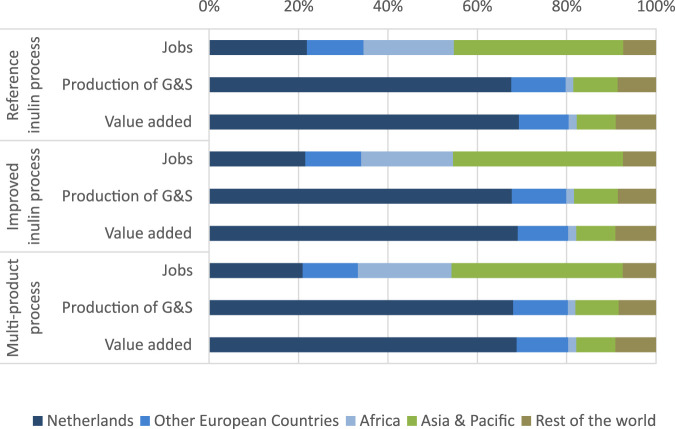
Distribution of indirect and induced impacts across regions.

In order to study in more detail the differences in impacts between the scenarios, we calculate final demand multipliers (see Table 3). Multipliers are values that quantify the economic impacts derived from a perturbation on the system. These include the direct consequence caused by the initial effects as well as the indirect ripples of the total effects on the economy (Miller and Blair, 2009). The multiplier effect is the ratio between the total economic impact estimated, denoted as *X* in the previous equations and the amount of money directly spent in the NPBT scenarios in terms of goods and services, denoted as *y* in the equations (and reported as total final demand in Table 1). The highest multipliers are gained in the multi-product process; thus when the final consumer requires 1 € of inulin and terpenes sold at the gate of Sensus BV, this generates a value added of 2.4 € and there will be a total production of goods and services in the whole economy equivalent to 4.3 € (direct, indirect and induced). The respective multiplier in the reference scenario and in the scenario with improved inulin process are slightly smaller. These results highlight the ability of chicory based inulin and terpenes to boost economic activity and increase competiveness of the European Union. The latter is also underscored by the regional allocation of impacts (see Figure 4). Not surprisingly, the highest impacts are found for the Netherlands. With a share of around 70% in case of value added and production of goods and services the impact is quite high. Due to strong EU-wide trade linkages, other European countries also profit. In total, nearly 80% of the gained value added and production output remain to the EU. However, turning to number of jobs we find that due to the comparably extremely low wages in Africa as well as South-East Asia in combination with the high importance of the indirect impact channel, these regions show the highest employment effects.

**FIGURE 5:**
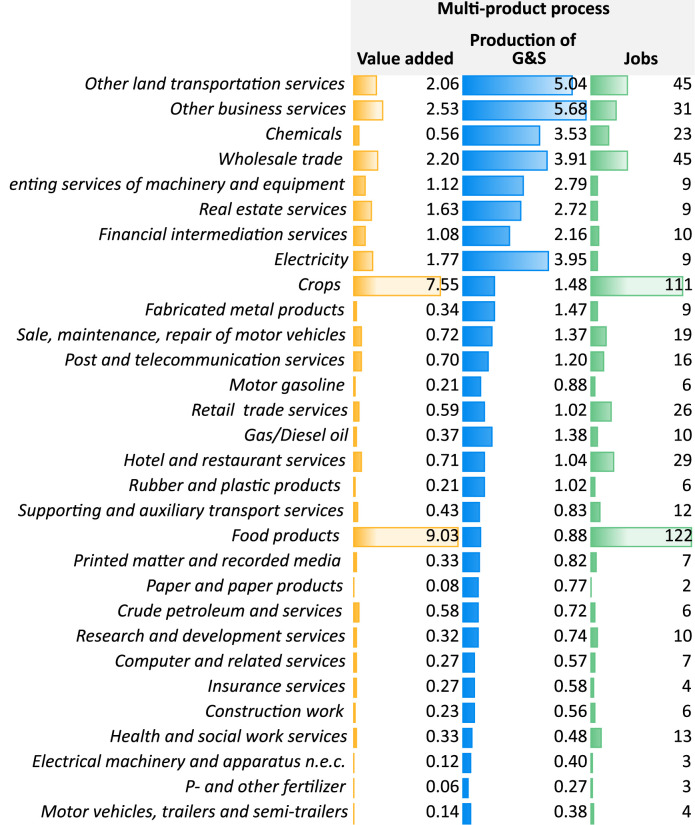
Impacts of value added, production of goods and services (G&S) and jobs on economic sectors (Top-30 ranked sectors are illustrated).

One of the main advantages of MRIO analysis is not only to identify regions in which impacts occur but also the activity sectors with the highest contributions. Since direction of effects is similar in all scenarios, Figure 5 illustrates impacts on economic sectors on the example of the multi-product process (sectorial impacts on the two remaining scenarios are reported in Supplementary Appendix Figure SA1). Impacts on economic sectors depend on the indicator. Regarding value added and employment, the food products, crops, transport services, other business services and wholesale trade sectors benefit the most. The latter three also rank highest in terms of production output y . The results show that key service sectors, such as business services, wholesale trade, renting of machinery and telecommunication as well as manufacturing industries such as chemicals, electricity and metal are highly interconnected in the economy and thus benefit in all scenarios. The production of medicinal terpenes makes alternative input goods available to the medicinal and pharmaceutical industry—included in Other Services. Possible substitution effects were not considered in this analysis due to lacking information about new processes or products. On a regional perspective, the Netherlands are mainly responsible for the socio-economic impacts regarding value added and production output. In terms of employment, the impacts on number of jobs stem to a large degree from the Asian & Pacific region (on country level employment is most stimulated in China, India and Indonesia).

### Environmental impacts

**FIGURE 6:**
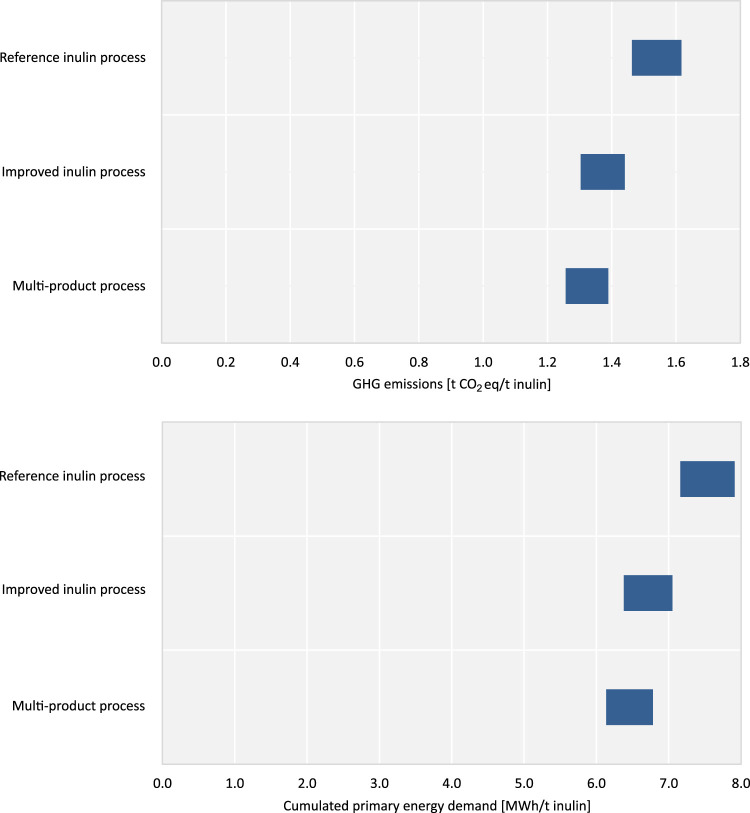
GHG emissions and cumulated primary energy demand of the new scenarios in comparison to the reference inulin production.

Using LCA, the impacts on GHG emissions and cumulated primary energy demand of the whole value chain are quantified. Figure 6 shows the GHG emissions and cumulated primary energy demand of the analyzed NPBT scenarios in comparison to the reference scenario. The environmental impacts of the multi-product process are allocated to t inulin produced to enable a comparison (see Section Scenarios). The highest GHG emissions show the reference inulin process with 1.46–1.62 t CO_2_eq/t inulin, followed by the improved inulin process with 1.30–1.44 t CO_2_eq/t inulin and the multi-product process with 1.26–1.39 t CO_2_eq/t inulin. In relation to the cumulated primary energy demand, also the reference inulin process shows the highest amount with 7.16–7.91 MWh/t inulin, followed by the improved inulin process with 6.38–7.05 MWh/t inulin and the multi-product process with 6.14–6.78 MWh/t inulin. The higher inulin content in the chicory roots is important for the reduction of GHG emissions and cumulated primary energy demand of the NPBT scenarios. This effect is most visible by the comparison of the improved inulin process with the reference inulin process. While the improved inulin process show lower GHG emissions and the cumulated primary energy demand per t inulin by approx. 11% (0.15–0.18 t CO_2_eq/t inulin; 0.78 to 0.86 MWh/t inulin), within the multi-product process we see a reduction of approx. 14% (0.21–0.23 t CO_2_eq/t inulin; 1.02 to 1.13 MWh/t inulin) in comparison to the reference inulin process. The fossil primary energy demand contributes between 71% in the improved inulin process (4.55–5.03 MWh/t inulin) and the reference inulin process (5.11–5.64 MWh/t inulin), and 75% (4.60–5.08 MWh/t inulin) in the multi-product process to the total cumulated primary energy demand; the renewable energy demand between 22% (1.38–1.52 MWh/t inulin) in the multi-product process and 26% in the improved inulin process (1.63–1.81 MWh/t inulin) and the reference inulin process (1.83–2.03 MWh/t inulin), and the other primary energy demand by approx. 3% (0.16–0.24 MWh/t inulin) in all the scenarios. The multi-product process is characterized by a higher fossil primary energy demand, since the production process requires a higher amount of natural gas per t inulin.

**FIGURE 7:**
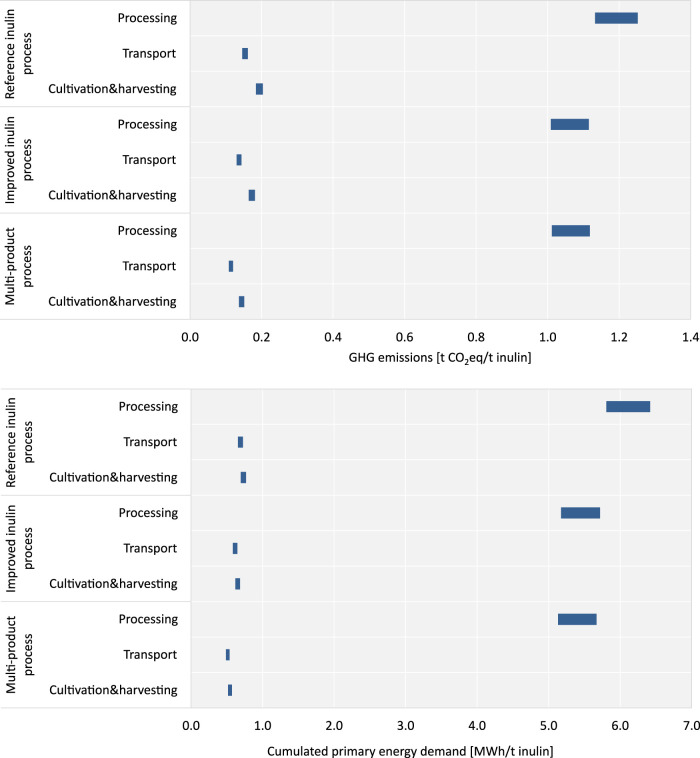
Contributions to the GHG emissions and cumulated primary energy demand by process steps (cultivation and harvesting, transport and processing).

Focusing on the three main process steps (cultivation and harvesting, transport as well as processing of inulin), reveals, as indicated in Figure 7, that the highest contribution to the total GHG emissions (1.01–1.25 t CO_2_eq/t inulin, resp. 77%–80%) and the cumulated primary energy demand (5.13–6.42 MWh/t inulin, resp. 81%–84%) stems from the processing of the chicory roots to inulin. In contrast, the process step of transport contributes the smallest to the environmental impacts (0.11–0.16 t CO_2_eq/t inulin, resp. 9%–10% of the total GHG emissions, 0.49 to 0.73 MWh/t inulin, resp. 8%–9% of the total primary energy demand) in all scenarios. Due to the higher inulin content in the roots in the two NPBT scenarios, GHG emissions and primary energy demand are significantly smaller compared to the reference inulin process. This trend is observed in all process steps. However, comparing GHG emissions and cumulated primary energy demand of both NPBT scenarios reveals a similar magnitude with only negligible differences.

**FIGURE 8:**
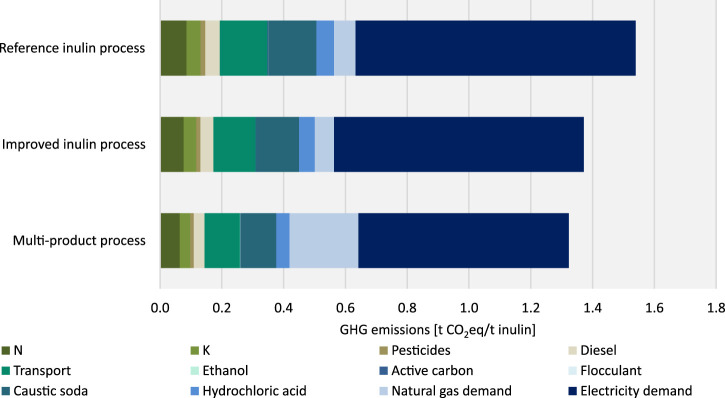
GHG emissions by auxiliary materials and auxiliary energy in cultivation and harvesting, transport and processing.

Figure 8 shows the contributions of the different auxiliary materials and auxiliary energy required in the process steps of cultivation and harvesting and transport from the field to the factory. Therein the electricity mix is the dominant source of GHG emissions. The share of GHG emissions from electricity amounts to approx. 59% of the total GHG emissions in case of the improved inulin process (0.81 t CO_2_eq/t inulin) and the reference inulin process (0.91 t CO_2_eq/t inulin). The multi-product process shows with approx. 51% (0.68 t CO_2_eq/t inulin) the smallest but still substantial contribution of the electricity mix of the total GHG emissions. In contrast, the share of natural gas of the total GHG emissions is highest in the multi-product process (approx. 17%, 0.22 t CO_2_eq/t inulin), while it is significantly lower in the other two scenarios (approx. 5%, 0.06 resp 0.07 t CO_2_eq/t inulin). N_2_O emissions from the direct use of nitrogen fertilizer on the field are included using IPCC guidelines (IPCC, 2019). Direct nitrogen-fertilizer induced emissions in form of N_2_O are contributing by about 2% to total GHG emissions. In terms of shares of GHG emissions, the reference inulin process and the improved inulin process do not show any significant differences, however, in absolute numbers GHG emissions are much lower in the improved inulin process compared to the reference inulin process.

## Discussion and conclusion

Despite the relevance of inulin with respect to human health and the significant market value, chicory is still an underdeveloped crop on the available arable land in Europe. One reason is that other crops are more beneficial, in terms of earnings, for the farmers. New plant breeding technologies developed in the CHIC project (H2020-NMBP-BIOTEC-07-2017, GA No. 760891) result in chicory with a reduced stress reaction and as a result of that a higher overall inulin yield per year and additionally extract terpenes, health-related compounds, thus enabling higher productivity compared to conventional chicory (Matos et al., 2020; Baixinho et al., 2021; Cankar et al., 2021; Häkkinnen et al., 2021; Matos et al., 2021) and increasing the competitive value of chicory in the rotation cycle of the farmer. Two scenarios based on new chicory variants are analyzed and impacts thereof are compared to the reference scenario, which describes the current commercial inulin process from conventional chicory. Both NPBT scenarios show a higher overall inulin content resulting in a higher inulin yield per year, but the inulin adsorption process differs. While one aims to optimize overall inulin yield, the other one explores the potential of a multipurpose use, yielding inulin and health beneficial terpenes.

By conducting an environmental and socio-economic impact assessment of the whole value chain of the new chicory variants and their derived products, we find that NPBTs have the potential to 1) boost economic activity as well as competiveness and 2) reduce primary energy demand and lower GHG emissions.

More precisely, by applying multi-regional input output analysis, we find that inulin from new chicory variants creates more jobs and generates higher value added as well as production from goods and services throughout the whole economy, as the current commercial process based on conventional chicory. The impact of reduced inulin breakdown (and thus an overall higher inulin content) has a significant positive impact on socio-economic indicators. The highest impacts are gained in the multi-product process, where additional value added compared to the reference inulin process amounts to over 40 million EUR. The production of goods and services as well as the value added increase with 5 million EUR for the improved inulin process. What can also be seen, is that the value added of the multi-product process and thus also commercializing health promoting terpenes contributes even more (16 million EUR) in relation to the reference inulin production. Although not compared directly in this paper, it could be argued that commercializing the terpenes for the current crop without any breeding optimization would also be commercially attractive. From a regional perspective, nearly 70% of the generated value added accrue within Netherlands and over 80% are generated within Europe. Outside Europe, due to import leakage, Chinese and United States sectors are the most to benefit. Generally, the sectors most stimulated by the new processes are agricultural and food production, chemical sector, business services and transport. From a policy perspective, the ability of NPBT based value chains to improve EUs competitiveness and reduce foreign dependency requires an exploitation of their potential in regional and EU-wide food policy strategies.

In terms of environmental impacts, life cycle analysis (LCA) underscores how the inulin process based on new chicory variants leads to lower GHG emissions and cumulated primary energy demand in comparison to the reference inulin process. The main reason, as in the socio-economic assessment, for this positive effect of the NPBT scenarios roots in inulin content per t chicory. GHG emission reduction ranges between 11% and 14%, depending on the adopted process. Additional, regarding climate neutral production, we find that a reduction by 47%–53% of the total GHG emissions of the scenarios is possible by using a renewable electricity mix to cover the electricity demand in the processing step instead of the national electricity mix. The cumulated primary energy demand could be reduced by 22%–26% applying a renewable electricity mix. The national electricity mix of the Netherlands is relatively GHG intense since generation has a high proportion of natural gas (36%) and coal (18%) (European Commission, 2020).

Summarizing, from a socio-economic and environmental point of view, the multi-product process, is the most beneficial one. This is not surprising, given the fact that this process yields two products, inulin and terpenes. Still, also the improved inulin process generates positive economic effects and leads to lower GHG emissions as well as primary energy demand compared to the reference inulin process. On a broader perspective, in addition to the results gathered in this case study, it can be envisioned that the use of NPBT’s in chicory will impact human health by providing established health promoting ingredient like inulin more efficiently, but also to enable the production of new health promoting ingredients such as STL’s. The results of this study confirm that this crop improvement has a much broader positive impact through the whole production cycle starting with an improved competitiveness of chicory in the rotation cycle of the European farmer and resulting in broader positive economic and environmental effects for Europe.

It must be noted that both of the assessments have limitations. The accuracy of the impact assessment depend strongly on the quality of the input data. The modelled data for this case study are based on a research project. The assessment of the case study builds on the best data available. They are consistent across the scenarios compared. Still, in order to tackle the limitation of modelled data, we conducted robustness checks by means of sensitivity analysis and crosschecked with relevant literature. Sensitivity analysis by varying the inulin content (15%–21%) and the productivity (increasing and decreasing the chicory yield per ha) in the NPBT scenarios support our findings and still shows that in most settings, NPBT processes generate higher economic impacts. Similarly, assuming a higher yield of the new chicory variants per hectare by 50 t chicory roots per year instead of 46 t chicory roots per year, the total GHG emissions could be reduced by approx. 1%, while a lower yield by 40 t chicory roots per ha and year would raise the GHG emissions by approx. 2%. Future research could use data from successful field trials to evaluate the potential of NPBT based value chains to contribute to the goal of a fair, sustainable, healthy and environmentally friendly food system in Europe and to derive specific up-scaled scenarios to meet this objective.

LCA and socio-economic assessments contribute to understand not only the direct effects of a whole process, but also indirect and induced effects resulting from different products or processes. Although the socio-economic and environmental impacts estimated in this research reflect a very specific case study, and should be treated accordingly, these results could easily be transferred to other crops used for dietary fibers and nutraceuticals in Central Europe with similar agriculture cycle and therefore similar cost structure.

## Data Availability

The original contributions presented in the study are included in the article/Supplementary Material, further inquiries can be directed to the corresponding author.

## Footnotes

1 The debate on the legal status of genome editing in the EU is ongoing and uncertain. The current EU legislation has clear implementation challenges and is not fit for purpose for some new genome techniques. The European Commission (European Commission, 2021) recommends that it needs to be revised to scientific as well as technological progress; in particular in the light of The European Green Deal, the Farm-to-Fork and the biodiversity strategy.

2 Note that these differences due to the respective requirements of the socio-economic and environmental assessment, have no impact on the results and conclusions drawn.
