# Supplementary material for: Environmental and socio-economic impacts of new plant breeding technologies: A case study of root chicory for inulin production
Source: Front Genome Ed. 2022 Oct 6;4:919392. doi: 10.3389/fgeed.2022.919392 (PMC9582860; doi:10.3389/fgeed.2022.919392)
Supplement: Supplementary file 1 [file Table1.docx]

Table SA1 Characteristics of the two NPBT scenarios

|  | Improved inulin process | Multi-product process |
| --- | --- | --- |
| **Plant defined as GMO due to ruling** | No – new breeded chicory variant without terpene accumulation | No – new breeded chicory variant |
| **Cultivation** | Open field | Open field |
| **Process optimized for** | Inulin production | Multi-product process |
| **Products** | Inulin (food) | Inulin (food) + Terpenes (nutraceutical) |
| **Further characteristics** | Produced with an adapted current commercial process as no terpenes needs to be extracted |  |
