# Supplementary material for: Environmental and socio-economic impacts of new plant breeding technologies: A case study of root chicory for inulin production
Source: Front Genome Ed. 2022 Oct 6;4:919392. doi: 10.3389/fgeed.2022.919392 (PMC9582860; doi:10.3389/fgeed.2022.919392)
Supplement: Supplementary file 2 [file Table2.docx]

Table SA2 Cost vector of each scenario by economic sector

|  | Reference inulin process | Improved inulin process | Multi-product process |
| --- | --- | --- | --- |
|  | *In million €* | | |
| **Agriculture** | **0.64** | **0.71** | **0.71** |
| Crops | 0.64 | 0.71 | 0.71 |
| **Manufacturing** | **0.25** | **0.26** | **0.53** |
| Paper and paper products | 0.21 | 0.22 | 0.31 |
| Printed matter and recorded media | 0.14 | 0.14 | 0.2 |
| N-fertilizer | 0.11 | 0.11 | 0.11 |
| P- and other fertilizer | 0.12 | 0.12 | 0.12 |
| Fabricated metal products | 0.34 | 0.36 | 0.5 |
| Machinery and equipment | 0.05 | 0.06 | 0.08 |
| Office machinery and computers | 0.05 | 0.05 | 0.07 |
| Electrical machinery and apparatus | 0.06 | 0.06 | 0.09 |
| **Fossil fuels** | **0.25** | **0.26** | **0.53** |
| Gas/Diesel oil | 0.08 | 0.08 | 0.36 |
| Motor gasoline | 0.17 | 0.17 | 0.17 |
| **Chemicals and plastics** | **1.45** | **1.49** | **1.9** |
| Chemicals | 1.22 | 1.26 | 1.27 |
| Rubber and plastic products | 0.24 | 0.25 | 0.35 |
| Plastics, basic | 0 | 0 | 0.4 |
| **Electricity** | **1.51** | **1.61** | **1.63** |
| Electricity | 1.51 | 1.61 | 1.63 |
| **Trade & repair services** | **1.43** | **1.53** | **2.09** |
| Sale, maintenance, repair of motor vehicles | 0.27 | 0.29 | 0.4 |
| Wholesale trade | 0.9 | 0.95 | 1.31 |
| Retail trade services | 0.27 | 0.28 | 0.39 |
| **Transport** | **2.06** | **2.06** | **2.08** |
| Other land transportation services | 1.99 | 1.99 | 1.99 |
| Supporting and auxiliary transport services | 0.06 | 0.07 | 0.09 |
| **Other services** | **2.59** | **2.76** | **3.56** |
| Post and telecommunication services | 0.06 | 0.06 | 0.09 |
| Financial intermediation services | 0.2 | 0.22 | 0.29 |
| Real estate services | 0.1 | 0.1 | 0.14 |
| Renting services of machinery and equipment | 0.9 | 0.92 | 1.07 |
| Computer and related services | 0.09 | 0.1 | 0.13 |
| Research and development services | 0.12 | 0.18 | 0.23 |
| Other business services | 1.1 | 1.17 | 1.61 |
| Membership organisation services | 0.01 | 0.01 | 0.01 |
| **Total final costs** | **11.01** | **11.56** | **14.07** |
