# Supplementary material for: Environmental and socio-economic impacts of new plant breeding technologies: A case study of root chicory for inulin production
Source: Front Genome Ed. 2022 Oct 6;4:919392. doi: 10.3389/fgeed.2022.919392 (PMC9582860; doi:10.3389/fgeed.2022.919392)
Supplement: Supplementary file 3 [file Table3.docx]

Table SA3 Life Cycle Inventory

|  |  | Unit | Reference inulin process | Improved inulin process | Multi-product process |
| --- | --- | --- | --- | --- | --- |
| **Cultivation & harvesting** | | | | | |
|  | Agricultural area | [ha] | 1,463 | 1,304 | 1,304 |
| Auxiliary materials | |  |  |  |  |
|  | N | [t N/a] | 102 | 91 | 91 |
|  | K | [t K_2_O/a] | 263 | 235 | 235 |
|  | Pesticides | [t /a] | 15 | 14 | 14 |
| Auxiliary energy | |  |  |  |  |
|  | Diesel | [l/a] | 174,072 | 155,222 | 155,222 |
| Product |  |  |  |  |  |
|  | Chicory plants harvested | [t/a] | 67,288 | 60,002 | 60,002 |
|  | Chicory plants harvested | [t_DM_/a] | 16,889 | 15,060 | 15,060 |
|  | Water content | [%] | 75 | 75 | 75 |
| **Transport** | | | | | |
|  | Average distance from field to factory | [km] | 100 | 100 | 100 |
| **Processing** | | | | | |
| Input |  |  |  |  |  |
|  | Chicory plants harvested | [t/a] | 67,288 | 60,002 | 60,002 |
|  | Chicory plants harvested | [t_DM_/a] | 16,889 | 15,060 | 15,060 |
|  | Water content | [%] | 75 | 75 | 75 |
|  | Inulin content | [%] | 17 | 19 | 19 |
|  | Terpene content | [%] |  |  | 13 |
| Auxiliary materials | |  |  |  |  |
|  | EtOH | [t/a] | 0 | 0 | 13 |
|  | Active carbon | [t/a] | 9 | 4 | 4 |
|  | Flocculant | [t/a] | 5 | 4 | 4 |
|  | Caustic soda | [t/a] | 1,273 | 1,135 | 1,135 |
|  | Hydrochloric acid | [t/a] | 1043 | 930 | 930 |
| Auxiliary energy | |  |  |  |  |
|  | Natural gas demand | [Nm³/a] | 313,676 | 279,708 | 1,201,533 |
|  | Electricity demand | [MWh/a] | 21,196 | 18,901 | 19,076 |
| Products |  |  |  |  |  |
|  | Inulin | [t/a] | 10,619 | 10,619 | 10,619 |
|  | Inulin | [t_DM_/a] | 10,300 | 10,300 | 10,300 |
|  | Water content | [%] | 3 | 3 | 3 |
|  | Terpenes | [t/a] | 0 | 0 | 2,111 |
|  | Terpenes | [t_DM_/a] |  |  | 1,056 |
|  | Ton active ingredient | [t/a] |  |  | 38 |
|  | Water content | [%] |  |  | 50 |
