# Supplementary figures and images for: Environmental and socio-economic impacts of new plant breeding technologies: A case study of root chicory for inulin production
Source: Front Genome Ed. 2022 Oct 6;4:919392. doi: 10.3389/fgeed.2022.919392 (PMC9582860; doi:10.3389/fgeed.2022.919392)

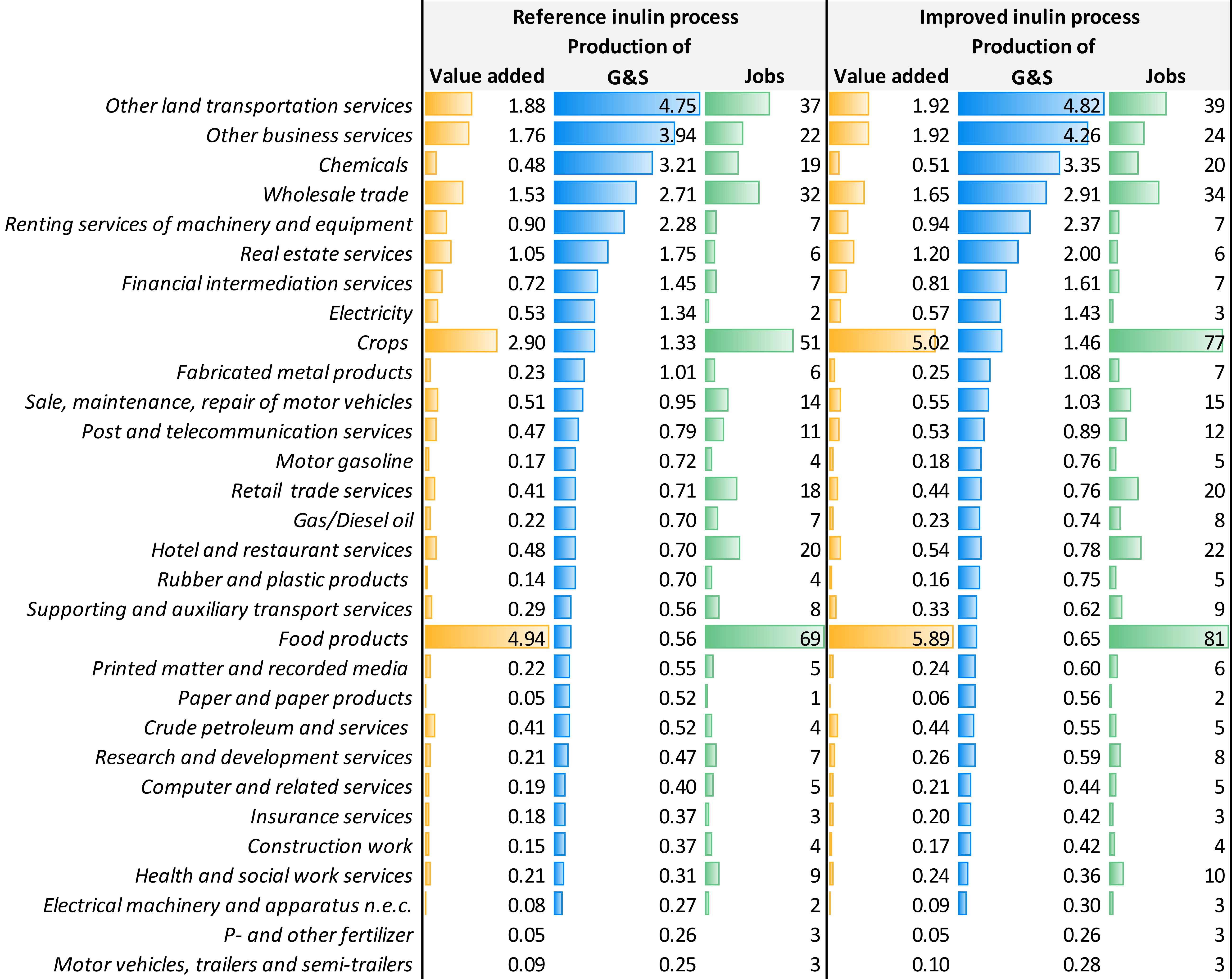

Supplement: Supplementary file 4 [file Image1.jpg]
